# Risk of death from cardiovascular disease following breast cancer: a systematic review

**DOI:** 10.1007/s10549-017-4282-9

**Published:** 2017-05-13

**Authors:** S. A. M. Gernaat, P. J. Ho, N. Rijnberg, M. J. Emaus, L. M. Baak, M. Hartman, D. E. Grobbee, H. M. Verkooijen

**Affiliations:** 1 Department of Epidemiology, University Medical Center Utrecht, Julius Center for Health Sciences and Primary Care, 3584 CX Utrecht, The Netherlands; 2 National University of Singapore, Saw Swee Hock School of Public Health, Singapore, 117549 Singapore; 3 Division of Internal Medicine, Academic Medical Center, 1105 AZ Amsterdam, The Netherlands; 4 Imaging Division, University Medical Center Utrecht, 3584 CX Utrecht, The Netherlands; 5 Utrecht University, 3584 CX Utrecht, The Netherlands; 6 Department of Surgery, National University Hospital Singapore, Singapore, 119074 Singapore

**Keywords:** Breast cancer, Cardiovascular disease, Absolute risk, Risk factors

## Abstract

**Purpose:**

Breast cancer incidence and survival is high, which results in high prevalence of breast cancer survivors. The risk of (death from) cardiovascular disease (CVD) is higher in patients exposed to cardiotoxic treatments, in particular if they have pre-existing CVD risk factors. This study systematically summarized the risk of death from CVD following breast cancer.

**Methods:**

Databases of Medline, Embase, and the Cochrane Library were systematically searched using the following terms and synonyms: breast cancer, cardiovascular disease, and cause of death. Articles reporting on both risk and risk factors of CVD mortality following breast cancer were eligible for inclusion. The methodological quality of each article was assessed using the Newcastle Ottawa quality assessment scale for cohort studies.

**Results:**

Fourteen articles were included assessing the risk of CVD mortality among 1,217,910 women with breast cancer. The methodological quality was high for the majority of the studies. Studies were heterogeneous in design, study population, length of follow-up, CVD outcomes, and risk factors. 1.6–10.4% of all women with breast cancer died of CVD. Women with breast cancer had a higher risk of CVD mortality than women from the general population. The risk of CVD mortality was higher among women with breast cancer with older age at diagnosis, left-sided tumor, diagnosis in an earlier calendar period, and black ethnic origin.

**Conclusions:**

CVD is an important cause of death following breast cancer. Identification of patients at high risk of CVD is important to optimize CVD prevention and tailor breast cancer treatment.

## Introduction

Breast cancer incidence has increased substantially over the last decades [1, 2], which, in combination with improved survival rates attributable to the availability of screening methods and effective treatments of early and more advance breast cancer [3, 4], leads to an increasing number of breast cancer survivors. Cardiovascular disease (CVD) is an important cause of death among these women as the risk of CVD may be increased by cardiotoxic treatments and CVD risk factors [5–8].

The risk of (death from) CVD following breast cancer is increased in women exposed to cardiotoxic treatments such as mediastinal and left-sided radiotherapy, anthracycline-based chemotherapy, and trastuzumab, and is even higher in patients with pre-existing CVD risk factors such as diabetes and hypertension [9–12]. With the current high breast cancer survival rates, especially for women with lower stages, and the large number of women with breast cancer receiving intensive treatment regimens, it is increasingly important to identify patients at high risk of CVD, and to balance the benefits of breast cancer treatment for achieving tumor control with the risks of cardiac toxicity inducing CVD.

As an overview of the available evidence on the risk of dying of CVD in women with breast cancer is currently lacking, we systematically reviewed the literature on the risk and risk factors of death from CVD following breast cancer.

## Methods and materials

This systematic review was conducted in accordance with the Preferred Reporting Item for Systematic Reviews and Meta-Analyses (PRISMA) guidelines [13].

A systematic search was performed, and last updated on April 1, 2017, to identify all studies reporting on the risk and risk factors of death from CVD following breast cancer. Databases of Medline (via PubMed), Embase, and the Cochrane Library were systematically searched using the following terms and their synonyms in the search strategy: breast cancer, cardiovascular disease, and cause of death (Table 1). No limits were used.

**Table 1:** Search strategy performed in Medline (via Pubmed)

	Search strategy (Medline via Pubmed)
#1	(Breast Neoplasms[Mesh Terms] OR cancer[Title/Abstract] OR cancers[Title/Abstract] OR carcinoma[Title/Abstract] OR carcinomas[Title/Abstract] OR tumor[Title/Abstract] OR tumors[Title/Abstract] OR tumor[Title/Abstract] OR tumors[Title/Abstract] OR malignancy[Title/Abstract] OR malignancies[Title/Abstract] OR neoplasm[Title/Abstract] OR neoplasms[Title/Abstract] OR neoplasms[Mesh Terms]) AND (breast[Title/Abstract] OR breasts[Title/Abstract] OR mamma[Title/Abstract] OR mamma*[Title/Abstract])
#2	(Cardiovascular Diseases[Mesh] OR heart[Title/Abstract] OR cardiac[Title/Abstract] OR cardio[Title/Abstract] OR cardiovascular[Title/Abstract] OR coronary[Title/Abstract] OR ventricular[Title/Abstract] OR valvular[Title/Abstract] OR circulatory[Title/Abstract]) AND (disease[Title/Abstract] OR diseases[Title/Abstract] OR complication[Title/Abstract] OR complications[Title/Abstract] OR failure[Title/Abstract] OR failures[Title/Abstract] OR dysfunction[Title/Abstract] OR dysfunctions[Title/Abstract] OR mortality[Title/Abstract] OR mortalities[Title/Abstract] OR death[Title/Abstract] OR deaths[Title/Abstract] OR arrhythmias[Title/Abstract] OR arrhythmia[Title/Abstract] OR cardiomyopathy[Title/Abstract] OR cardiomyopathies[Title/Abstract] OR Ischemia[Title/Abstract] OR Ischemia’s[Title/Abstract] OR all[Title/Abstract]) AND (cause[Title/Abstract] or causes[Title/Abstract] OR other[Title/Abstract])
#3	(Cause of death[Mesh Terms] OR mortality[Title/Abstract] OR mortalities[Title/Abstract] OR death[Title/Abstract] OR deaths[Title/Abstract] OR fatality[Title/Abstract] OR fatalities[Title/Abstract] OR dying[Title/Abstract])
#4	#1 AND #2 AND #3

Comparable search strategies have been conducted for Embase and the Cochrane Library

Articles reporting on both risk and risk factors of CVD mortality in breast cancer patients were eligible for inclusion. Articles with the following criteria were excluded: (1) published before 1990, (2) written in another language than English or Dutch, (3) case reports, reviews, or abstracts. Cross-referencing was performed.

### Selection of studies and data extraction

After removal of duplicates, all titles and abstracts of the remained retrieved articles were screened. Abstracts that seemed potentially relevant, based on the in- and exclusion criteria, were screened for full text. The full text of these articles were assessed for eligibility by three investigators independently (S.A.M. Gernaat, P.J. Ho, and N. Rijnberg). Data were extracted using standardized data extraction forms and any disagreements were resolved by discussion. We extracted data on study size, characteristics of breast cancer patients (age, ethnic origin, year of diagnosis, years of follow-up), study design, International Classification of Diseases (ICD) codes for CVD mortality, absolute risk of death from CVD, absolute risk of death from breast cancer, absolute risk of death from any cause, statistical methods used to assess which factors increase the risk of death from CVD, and the risk of CVD mortality per risk factor.

### Quality assessment

The methodological quality for each article was assessed by two authors independently (S.A.M. Gernaat and P.J. Ho) using the Newcastle Ottawa Quality Assessment Scale (NOS) for cohort studies [14]. The NOS consists of six multiple-choice questions that address subject selection, comparability, and the assessment of the outcome (i.e. CVD mortality), which sum up to a maximum score of seven. In the present study, a high score on one of these sections indicated that the maximum score (i.e. two for selection and comparability, and three for outcome) was achieved. In all other cases, the study received a low score on that particular section.

## Results

**Fig. 1:**
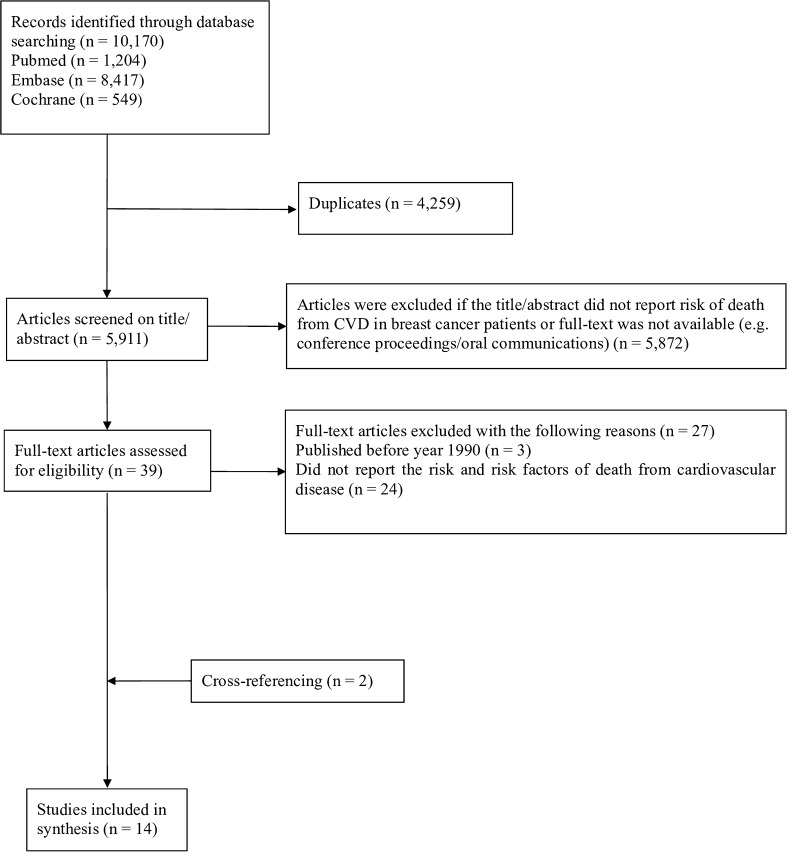
Flowchart of the systematic review on the risk of death from cardiovascular disease in breast cancer patients

The systematic search yielded 10,170 citations including 5911 unique articles, which were screened for title and abstract using the predefined inclusion and exclusion criteria (Fig. 1). After screening the full text of 39 articles, 27 were excluded for the following reasons: published before the calendar year 1990 (*n* = 3) or articles that did not report the risk and risk factors of death from CVD (*n* = 24). Cross-referencing identified two additional papers. In total, 14 articles were included in the current systematic review, including 4,773,576 women of which 1,217,910 were diagnosed with breast cancer [5, 6, 8, 15–25].

### Quality assessment

**Fig. 2:**
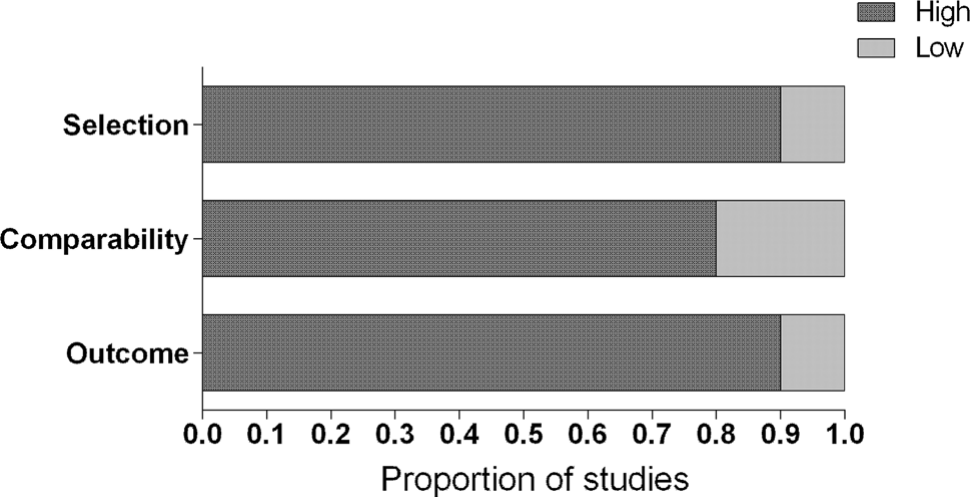
Quality assessment by the Newcastle Ottawa Quality Assessment Scale Selection was based on the representativeness of the breast cancer cohort and ascertainment of breast cancer. Comparability was based on the comparability of cohorts on the basis of the design or analysis. Outcome was based on the assessment of death from cardiovascular disease, on the length of follow-up (≥10 years), and adequacy of follow-up of the cohorts. A high score on one of these sections indicated that the maximum score on that particular section (i.e. two for selection and comparability, and three for outcome) was achieved. In all other cases, the study received a low score on that particular section

The majority of the studies had the maximum score on the quality assessment for selection, comparability, and outcome [5, 8, 17, 18, 20–22, 26] (Fig. 2). The study by Nichols et al. [15] had a low score on selection as the study population was a selected group of in situ or invasive breast cancer patients and breast cancer ascertainment was by written self-report. The studies by Berkman et al. [6], Darby et al. [23], and Giordano et al. [24] had low scores on comparability as the hazard ratios (HRs) were not adjusted for factors other than age at diagnosis, the CVD mortality rates were unadjusted, and the HRs were only adjusted for other factors than age at diagnosis, respectively. The studies by Hooning et al. [25] and McCullough et al. [16] had low scores on the outcome attainment as the assessment of CVD deaths was by hospital records and subjects were lost to follow-up or the follow-up rate was less than 70%, respectively.

### Cardiovascular disease mortality in breast cancer patients compared with the general population

Bradshaw et al. [8] included 1413 women with primary in situ or invasive breast cancer diagnosed in the United States (U.S.) between 1996 and 1997, and 1411 age-matched women from the general population (Table 2). Mean age at breast cancer diagnosis and reference date for women from the general population were 59 and 57 years, respectively. During the follow-up time, which ranged between 0.2 and 13.5 years for both groups, 9.4% of women with breast cancer and 7.4% of women from the general population died of CVD. After adjusting for age, menopausal status, and other CVD risk factors, women with breast cancer had a 1.9 (95% confidence interval (CI) = 1.4–2.7) times higher risk to die of CVD after at least 7 years post diagnosis than women from the general population (Table 3).

**Table 2:** Characteristics and risk of cardiovascular disease mortality of the fourteen articles included in the systematic review

First author, publication year, country	Type of breast cancer, number of patients	Age at diagnosis or reference date, y	Year of diagnosis, years of follow-up	ICD-9 and/or ICD-10 codes of CVD mortality outcomes	Percentage of deaths due to any cause, CVD, and BC (percentage of total)	
CVD mortality in breast cancer patients compared with the general population						
Bradshaw, 2016, US^a^	Primary in situ or invasive; 1413	59 or 57^d^	1996–1997, 13.5^e^	ICD-9: 394.9, 402.9, 410, 414.0, 427.5	*Women with BC*	*Women without BC*
					Any cause: 29.4	Any cause: 17.2
				ICD-10: I10, I11.9, I21.9, I25.1, I25.4, I46.9		
	Without BC; 1411^c^				CVD: 9.4 BC: 9.6	CVD: 7.4 BC: 0.1
Riihimäki, 2012, Sweden^a^	Primary invasive; 122,217	–	1987–2006, 19^e^	ICD-9: 410, 411–414, 420–427, 428, 430–438, 440–448	*Women with BC*	*Women without BC*
					Any cause: 39.3	Any cause: 16.7
	Women without BC; 3,554,255^h^					
				ICD-10: I20, I21–I22, I23–I25, I30–I50, I52, I60–I79	CVD: 10.4	CVD: 7.5
					BC: 18.1	BC: -
CVD mortality in breast cancer patients by patient, tumor, and treatment characteristics						
Colzani, 2011, Sweden^a^	Primary invasive I-III; 12,850	<75	1990–2006, 10^e^	ICD-9: 390–459	Any cause:	14.4
				ICD-10: I00–I99	CVD:	1.8
					BC:	9.2
Hooning, 2006, the Netherlands^f^	Primary invasive I-IIIA; 7425	≤70	1970–1986, 13.8^b^	ICD-9: 410–459	Any cause:	56.0
					CVD:	5.3
					BC:	42.6
CVD mortality in patients with left-sided breast cancer compared to right-sided breast cancer						
Bouchardy, 2009, Switzerland^a^	Primary invasive lymph node-negative; 1245	57.4^d^	1980–2004, 7.7^d^	ICD-10: I00-I99	Any cause:	12.4
					CVD:	2.2
					BC:	7.3
Darby, 2005, US^a^	Primary in situ or invasive; 308,861	20–79	1973–2001, 29^e^	ICD-9: 390–398, 402, 404, 410, 411–414, 415–429	Any cause:	29.5
					CVD:	4.2
					BC:	16.8
Giordano, 2005, US^a^	Primary in situ or invasive; 24,785	56.9 ± 13.2^d^	1973–1988, 9.3^b^	ICD-9: 410-414	Any cause:	–
				ICD-10: I20–I25	CVD:	–
					BC:	–
Haque, 2017, US^a^	DCIS, 140,914	≤60 & >60	1973–2002, 11.5	–	Any cause:	–
			(IQR: 6.8–15.1)^b^		CVD:	–
					BC:	–
Merzenich, 2016, Germany^g^	Primary in situ or invasive; 11,982	59^d^	1998–2008, 6.5 (0–15)^b^	ICD-10: I20–I25, I34–I37, I44–I50	Any cause:	20.6
		(range: 18–101)			CVD:	2.3
					BC:	10.2
CVD mortality in breast cancer patient with ethnic differences						
Berkman, 2014, US^a^	Primary DCIS; 54,518 white women; 6113 black women	≥40	1978–2010, 9.2^b^	ICD-10: I00–I09, I11, I13, I20–I51, I60–I69, I70, I72–178	Any cause:	18.0
					CVD:	6.0
					BC:	1.5
Solanki, 2016, US^a^	Primary in situ or invasive I-III; 462,005 NHW; 44,531 API	NHW: 61.2 ± 13.7^d^	1991–2011,	ICD-9: 390–459	*Non-Hispanic white*	*Asian and Pacific Islander*
		API: 56.3 ± 13.1^d^	NHW: 6.8 ± 4.9^d^,		Any cause: 23.8	Any cause: 15.4
			4 (2–6) ^b^		CVD: 5.5	CVD: 2.6
			API: 6.7 ± 5.0^d^,		BC: 10.0	BC: 8.2
			3 (2–5)^b^			
CVD mortality in breast cancer patients by diet, body weight, and health behaviors						
McCullough, 2016, Switzerland^g^	Primary invasive I-III; 4452 for pre-diagnostic of which 2152 were included in the ≥1-year post- diagnostic analysis	70.7 ± 7.2^d^	1992–2011, pre-diagnostic diet assessment 9.8 ± 4.9, post-diagnostic analyses 9.9 ± 3.3^d^	ICD-9: 390-459	Any cause:	27.0
				ICD-10: I00-I99	CVD:	5.2
					BC:	8.9
Nichols, 2009, US^a^	Primary in situ or invasive; 5791	58.4 ± 10.0^d^	1988–1999, 6.4 ± 1.2^d^	ICD-10: I00-99	Any cause:	7.3
					CVD:	1.6
					BC:	2.1
Veal, 2017, US^g^	Primary DCIS; 1925	20–74	1997–2006, 6.7^d^	ICD-10: I00-I09, I11, I13, I20-I51, I60-I69, I70, I72-I78	Any cause:	10.2
					CVD:	1.8
					BC:	4.5

*BC* breast cancer, *CVD* cardiovascular disease, *DCIS* ductal carcinoma in situ, *ICD-9* International Classification of Diseases version 9, *ICD-10* International Classification of Diseases version 10, *y* years, *US*  United States of America

^a^Population-based registry

^b^Median with (range if described by the article)

^c^Women without breast cancer were matched on age and the expected distribution of survivors in 5-year age groups with women with breast cancer

^d^Mean with (±standard deviation if described by the article)

^e^Maximum

^f^Hospital-based registry

^g^Prospective cohort study

^h^All women who were born before 1977 who resided in Sweden were included; women without breast cancer were part of the reference group

**Table 3:** Risk factors of death from cardiovascular disease in women diagnosed with breast cancer

First author, year of publication	Statistical analysis	Categories			Cause of death (outcome)	Risk of death HR (95% CI)	Covariates
CVD mortality in breast cancer patients compared with the general population							
Bradshaw, 2016	Competing risk	General population			CVD	1.0 (ref)	Age, menopausal status, CVD risk factors
		Breast cancer patients diagnosed 0–7 years ago				0.59 (0.4–0.9)	
		General population			CVD	1.00 (ref)	
		Breast cancer patients diagnosed >7 years ago				1.9 (1.4–2.7)	
		General population			CVD	1.0 (ref)	
		Breast cancer patients treated with RT				1.1 (0.8–1.6)	
		General population			CVD	1.0 (ref)	
		Breast cancer patients treated without RT				1.3 (0.9–2.0)	
		General population			CVD	1.0 (ref)	
		Breast cancer patients treated with CT				1.4 (1.0–2.2)	
		General population			CVD	1.0 (ref)	
		Breast cancer patients treated without CT				1.1 (0.8–1.5)	
		General population			CVD	1.0 (ref)	
		Breast cancer patients treated with HT				1.2 (0.9–1.7)	
		General population			CVD	1.0 (ref)	
		Breast cancer patients treated without HT				1.2 (0.8–1.8)	
Riihimäki, 2012	Cox proportional hazard	General population			CVA	1.00 (ref)	Age, socioeconomic index, geographical region of residence
		Breast cancer patients				1.03 (1.00–1.07)	
		General population			AMI	1.00 (ref)	
		Breast cancer patients				1.01 (0.98–1.0)	
		General population			Other CHD	1.00 (ref)	
		Breast cancer patients				1.14 (1.10–1.19)	
		General population			Heart failure	1.00 (ref)	
		Breast cancer patients				1.29 (1.22–1.37)	
		General population			Other heart disease	1.00 (ref)	
		Breast cancer patients				1.24 (1.17–1.32)	
		General population			Arterial disease	1.00 (ref)	
		Breast cancer patients				0.95 (0.89–1.02)	
		General population			Complications of CVD	1.00 (ref)	
		Breast cancer patients				1.12 (0.99–1.2)	
CVD mortality in breast cancer patients by patient, tumor, and treatment characteristics							
Colzani, 2011	Flexible parametric survival models	Age at diagnosis	<45		CVD	0.3 (0.0–2.5)	Clinical, tumor, treatment characteristics
			45–54			1.00 (ref)	
			55–64			6.5 (2.8–14.6)	
			65–74			17.9 (8.0–39.7)	
		Calendar time at diagnosis	1990–1994		CVD	2.1 (1.2–3.6)	
			1995–1999			1.6 (0.9–2.9)	
			2000–2006			1.00 (ref)	
		Treatment	Surgery		CVD	2.1 (1.2–3.8)	
			Surgery + RT + HT			1.00 (ref)	
			Surgery + RT			1.4 (0.7–2.5)	
			Surgery + RT + CT			0.6 (0.1–2.5)	
			Surgery + CT			2.0 (0.6–6.8)	
			Surgery + RT + CT + HT			0.7 (0.3–1.9)	
			Surgery + HT			2.2 (1.5–3.2)	
			Surgery + CT + HT			1.0 (0.2–4.5)	
		No. of positive lymph nodes	0		CVD	1.00 (ref)	
			1–3			2.0 (1.4–2.9)	
			≥4			(1.0-3.4)	
		Estrogen receptor	Negative		CVD	1.00 (ref)	
		Status	Positive			0.8 (0.5–1.3)	
		Tumor size in mm	1–20		CVD	1.00 (ref)	
			>20			1.5 (1.1–2.1)	
Hooning, 2006	Cox proportional hazard	*Total study population*					Clinical, tumor, treatment, characteristics
		Age at diagnosis (continuous)			CVD	1.12 (1.10–1.14)	
		Treatment	Surgery		CVD	1.00 (ref)	
			Surgery + RT			2.03 (1.33–3.10)	
			Surgery + RT + CT			1.47 (0.81–2.67)	
			Surgery + RT + HT			1.70 (0.99–2.93)	
		Calendar time at diagnosis	1970–1975		CVD	1.34 (0.93–1.92)	
			1976–1980			1.54 (1.11–2.14)	
			1981–86			1.00 (ref)	
		*10-year survivors*					
		Age at diagnosis (continuous)			CVD	1.11 (1.09–1.13)	
		Treatment	Surgery		CVD	1.00 (ref)	
			Surgery + RT			2.08 (1.25–3.47)	
			Surgery + RT + CT			2.38 (1.18–4.77)	
			Surgery + RT + HT			2.42 (1.27–4.61)	
		Calendar time at diagnosis	1970–1975		CVD	1.38 (0.89–2.14)	
			1976–1980			1.62 (1.07–2.46)	
			1981–1986			1.00 (ref)	
CVD mortality in breast cancer patients by laterality of the tumor							
Bouchardy, 2016	Cox proportional hazard	RT and right-sided tumor			CVD	1.00 (ref)	Clinical, tumor, and treatment characteristics
		RT and left-sided tumor				0.52 (0.24–1.12)	
		RT and outer quadrant			CVD	1.00 (ref)	
		RT and inner quadrant				2.46 (1.13–5.37)	
		RT and right-sided tumor and outer quadrant			CVD	1.00 (ref)	
		RT and right-sided tumor and inner quadrant				2.51 (0.88–7.18)	
		RT and left-sided tumor and outer quadrant			CVD	1.00 (ref)	
		RT and left-sided tumor and inner quadrant				2.17 (0.65–7.25)	
		RT and outer quadrant and right-sided tumor			CVD	1.00 (ref)	
		RT and outer quadrant and left-sided tumor				0.70 (0.21–2.32)	
		RT and inner quadrant and right-sided tumor			CVD	1.00 (ref)	
		RT and inner quadrant and left-sided tumor				0.52 (0.18-1.48)	
Darby, 2005	Poisson regression for mortality rates	RT on right-sided tumor			CVD	1.00 (ref)	No covariates
		RT on left-sided tumor				1.44 (1.26–1.65)	
		RT on right-sided tumor			AMI	1.00 (ref)	
		RT on left-sided tumor				1.43 (1.10–1.87)	
		RT on right-sided tumor			Other	1.00 (ref)	
					Ischemic		
					CVD		
		RT on left-sided tumor				1.60 (1.26–2.02)	
		RT on right-sided tumor and aged 20–49 years at diagnosis			CVD	1.00 (ref)	
		RT on left-sided tumor and aged 20–49 years at diagnosis				1.54 (1.08–2.19)	
		RT on right-sided tumor and aged 50–59 years at diagnosis			CVD	1.00 (ref)	
		RT on left-sided tumor and aged 50–59 years at diagnosis				1.53 (1.19–1.98)	
		RT on right-sided tumor and aged 60–69 years at diagnosis			CVD	1.00 (ref)	
		RT on left-sided tumor and aged 60–69 years at diagnosis				1.40 (1.15–1.70)	
		RT on right-sided tumor and aged 70–79 years at diagnosis			CVD	1.00 (ref)	
		RT on left-sided tumor and aged 70–79 years at diagnosis				1.28 (0.87–1.90)	
		RT on right-sided tumor and white ethnic origin			CVD	1.00 (ref)	
		RT on left-sided tumor and white ethnic origin				1.39 (1.21–1.61)	
		RT on right-sided tumor and black ethnic origin			CVD	1.00 (ref)	
		RT on left-sided tumor and black ethnic origin				2.25 (1.36–3.72)	
		RT on right-sided tumor and other/ unknown ethnic origin			CVD	1.00 (ref)	
		RT on left-sided tumor and other/ unknown ethnic origin				1.30 (0.71–2.39)	
Giordano, 2005	Cox proportional hazard	Right-sided tumor and diagnosed in 1979			CVD	1.00 (ref)	No covariates
		Left-sided tumor and diagnosed in 1979				1.50 (1.19–1.87)	
		Right-sided tumor and diagnosed in 1988			CVD	1.00 (ref)	
		Left-sided tumor and diagnosed in 1988				0.79 (0.52–1.18)	
Haque, 2016	Cox proportional hazard	*Diagnosed 1973–1982*					Clinical, tumor, treatment characteristics
		Right-sided tumor			CVD	1.00 (ref)	
		Left-sided tumor				1.30 (1.18–1.42)	
		Race	White		CVD	1.00 (ref)	
			African American			1.14 (0.94–1.36)	
			American Indian/			0.83 (0.66–1.02)	
			Asian/ Pacific				
			Islander				
			Unspecified			0.47 (0.03–2.06)	
		Age at diagnosis	≤60		CVD	1.00 (ref)	
			>60			5.87 (5.30–6.50)	
		Marital status	Married		CVD	1.00 (ref)	
			Unmarried			1.87 (1.70–2.05)	
			Unknown			1.77 (1.29–2.37)	
		Right-sided tumor and <10 years since diagnosis			CVD	1.00 (ref)	
		Left-sided tumor and <10 years since diagnosis				1.14 (0.99–1.32)	
		Right-sided tumor and 10–19 years since diagnosis			CVD	1.00 (ref)	
		Left-sided tumor and 10–19 years since diagnosis				1.32 (1.12–1.57)	
		Right-sided tumor and ≥20 years since diagnosis			CVD	1.00 (ref)	
		Left-sided tumor and ≥20 years since diagnosis				1.30 (1.10–1.54)	
		Region	Pacific		CVD	1.00 (ref)	
			Alaska			–	
			East			0.97 (0.86-1.09)	
			Northern Plains			1.35 (1.21-1.51)	
			Southwest			1.51 (0.98-1.34)	
		*Diagnosed 1983–1992*					
		Right-sided tumor			CVD	1.00 (ref)	
		Left-sided tumor				1.02 (0.95–1.10)	
		Race	White		CVD	1.00 (ref)	
			African American			1.14 (0.98–1.32)	
			American Indian/			0.68 (0.56–0.82)	
			Asian/ Pacific				
			Islander				
			Unspecified			0.39 (0.02–1.74)	
		Age at diagnosis	≤60		CVD	1.00 (ref)	
			>60			10.16 (9.62–11.30)	
		Marital status	Married		CVD	1.00 (ref)	
			Unmarried			2.25 (2.08–2.42)	
			Unknown			1.65 (1.28–2.08)	
		Right-sided tumor and <10 years since diagnosis			CVD	1.00 (ref)	
		Left-sided tumor and <10 years since diagnosis				1.01 (0.90–1.13)	
		Right-sided tumor and 10–19 years since diagnosis			CVD	1.00 (ref)	
		Left-sided tumor and 10–19 years since diagnosis				0.98 (0.88–1.10)	
		Right-sided tumor and ≥ 20 years since diagnosis			CVD	1.00 (ref)	
		Left-sided tumor and ≥20 years since diagnosis				0.94 (0.77–1.15)	
		Region	Pacific		CVD	1.00 (ref)	
			Alaska			0.00 (0.00–14.13)	
			East			1.09 (0.99–1.20)	
			Northern Plains			1.42 (1.30–1.56)	
			Southwest			1.13 (0.97–1.30)	
		*Diagnosed 1993–1902*					
		Right-sided tumor			CVD	1.00 (ref)	
		Left-sided tumor				0.99 (0.93–1.05)	
		Race	White		CVD	1.00 (ref)	
			African American			1.32 (1.20–1.45)	
			American Indian/			0.53 (0.46–0.61)	
			Asian/ Pacific				
			Islander				
			Unspecified			0.11 (0.01–0.47)	
		Age at diagnosis	≤60		CVD	1.00 (ref)	
			>60			10.73 (9.86–11.70)	
		Marital status	Married		CVD	1.00 (ref)	
			Unmarried			2.21 (2.28–2.55)	
			Unknown			1.90 (1.60–2.24)	
		Right-sided tumor and <10 years since diagnosis			CVD	1.00 (ref)	
		Left-sided tumor and <10 years since diagnosis				1.00 (0.98–1.03)	
		Right-sided tumor and ≥20 years since diagnosis			CVD	1.00 (ref)	
		Left-sided tumor and ≥20 years since diagnosis				1.01 (0.91–1.11)	
		Region	Pacific		CVD	1.00 (ref)	
			Alaska			0.24 (0.01–1.07)	
			East			1.06 (0.99–1.13)	
			Northern Plains			1.25 (1.16–1.35)	
			Southwest			0.87 (0.76–0.99)	
Merzenich, 2016	Cox proportional hazard	RT and right-sided tumor			CVD	1.0 (ref)	Clinical, tumor, treatment characteristics
		RT and left-sided tumor				0.94 (0.64–1.38)	
		No RT and right-sided tumor			CVD	1.0 (ref)	
		No RT and left-sided tumor				1.07 (0.79–1.46)	
		RT without a history of cardiac disease			CVD	1.0 (ref)	
		RT with a history of cardiac disease				1.73 (1.11–2.68)	
		RT without chemotherapy			CVD	1.0 (ref)	
		RT with chemotherapy				0.66 (0.37–1.19)	
CVD mortality in breast cancer patients with different ethnic origins							
Berkman, 2014	Kaplan– Meier with log-rank statistics	White and diagnosed between 1990 and 2010			CVD	1.00 (ref)	No covariates
		Black and diagnosed between 1990 and 2010				6.43 (3.61–11.45)	
		White and age at diagnosis 40–49 years			CVD	1.00 (ref)	
		Black and age at diagnosis 40–49 years				9.83 (4.56–21.17)	
		White and age at diagnosis 50–59 years			CVD	1.00 (ref)	
		Black and age at diagnosis 50–59 years				3.35 (2.14–5.24)	
		White and age at diagnosis 60–69 years			CVD	1.00 (ref)	
		Black and age at diagnosis 60–69 years				2.13 (1.65–2.74)	
		White and age at diagnosis ≥70 years			CVD	1.00 (ref)	
		Black and age at diagnosis ≥70 years				1.07 (0.93–1.23)	
Solanki, 2016	Cox proportional hazard	Non-Hispanic white			CVD	1.00 (ref)	Age, birthplace, SEER registry, AJCC stage
		Asian and Pacific islander				0.77 (0.71–0.83)	
		Non-Hispanic white			CVD	1.00 (ref)	
		Chinese				0.66 (0.56–0.78)	
		Non-Hispanic white			CVD	1.00 (ref)	
		Japanese				0.71 (0.62–0.81)	
		Non-Hispanic white			CVD	1.00 (ref)	
		Filipino				0.90 (0.78–1.03)	
		Non-Hispanic white			CVD	1.00 (ref)	
		Hawaiian				1.43 (1.17–1.75)	
		Non-Hispanic white			CVD	1.00 (ref)	
		Korean				0.68 (0.46–0.99)	
		Non-Hispanic white			CVD	1.00 (ref)	
		Vietnamese				0.46 (0.28–0.76)	
		Non-Hispanic white			CVD	1.00 (ref)	
		Asian Indian and Pakistani				0.98 (0.70–1.37)	
		Non-Hispanic white			CVD	1.00 (ref)	
		Pacific Islander				1.33 (0.83–2.15)	
		Non-Hispanic white			CVD	1.00 (ref)	
		Other Asian				0.61 (0.45–0.83)	
		Non-U.S. born Asian and Pacific Islander			CVD	1.00 (ref)	
		U.S. born Asian and Pacific Islander				1.29 (1.08–1.54)	
		Non-U.S. born Chinese			CVD	1.00 (ref)	
		U.S. born Chinese				1.33 (0.81–2.20)	
		Non-U.S. born Japanese			CVD	1.00 (ref)	
		U.S. born Japanese				1.04 (0.74–1.48)	
		Non-U.S. born Filipino			CVD	1.00 (ref)	
		U.S. born Filipino				0.99 (0.57–1.72)	
		Non-U.S. born Hawaiian			CVD	1.00 (ref)	
		U.S. born Hawaiian				0.97 (0.13–7.38)	
		Non-U.S. born Korean			CVD	1.00 (ref)	
		U.S. born Korean				0.17 (0.02–1.69)	
		Non-U.S. born Asian Indian and Pakistani			CVD	1.00 (ref)	
		U.S. born Asian Indian and Pakistani				0.94 (0.11–8.13)	
		Non-U.S. born Pacific Islander			CVD	1.00 (ref)	
		U.S. born Pacific Islander				4.27 (0.68–26.7)	
		Non-U.S. born Other Asian			CVD	1.00 (ref)	
		U.S. born Other Asian				2.06 (0.84–5.10)	
CVD mortality in breast cancer patients by diet, body weight, and health behaviors							
McCullough, 2016	Cox proportional hazard	Pre-diagnostic diet score (continuous)			CVD	0.96 (0.84–1.10)	Clinical, tumor, and treatment characteristics, CVD risk factors
		Pre-diagnostic diet score 0–2			CVD	1.00 (ref)	
		Pre-diagnostic diet score 3–5				0.95 (0.68–1.32)	
		Pre-diagnostic diet score 6–9				0.94 (0.63–1.39)	
		Post-diagnostic diet score (continuous)			CVD	0.95 (0.79–1.14)	
		Post-diagnostic diet score 0–2			CVD	1.00 (ref)	
		Post-diagnostic diet score 3–5				0.96 (0.60–1.54)	
		Post-diagnostic diet score 6–9				0.81 (0.47–1.39)	
Nichols, 2009	Cox proportional hazard	One to 5 year before diagnosis a BMI <18.5			CVD	4.15 (1.44–12.0)	Age, menopausal status, and other CVD risk factors
		One to 5 year before diagnosis a BMI 18.5−24.9				1.00 (ref)	
		One to 5 year before diagnosis a BMI 25.0−29.9				1.05 (0.63–1.74)	
		One to 5 year before diagnosis a BMI ≥30				2.45 (1.46–4.11)	
		BMI after diagnosis <18.5			CVD	0.58 (0.08–4.34)	
		BMI after diagnosis 18.5−24.9				1.00 (ref)	
		BMI after diagnosis 25.0−29.9				0.99 (0.59–1.66)	
		BMI after diagnosis ≥30				1.65 (0.97–2.83)	
		Weight (kg) change −50.0 to −10.1			CVD	1.08 (0.42–2.78)	
		Weight (kg) change −10.0 to −2.1				1.02 (0.58–1.80)	
		Weight (kg) change −2.0 to 2.0				1.00 (ref)	
		Weight (kg) change 2.1–6.0				0.79 (0.43–1.44)	
		Weight (kg) change 6.1–10.0				0.64 (0.29–1.44)	
		Weight (kg) change 10.1				1.73 (0.83–3.62)	
Veal, 2017	Cox proportional hazard	*Pre-diagnosis behaviors*					Demographic, clinical, tumor, and treatment characteristics
		BMI		Continuous	CVD	1.01 (0.95–1.07)	
				18.5–24.9	CVD	1.0 (ref)	
				25.0–29.9		0.88 (0.37–2.07)	
				30.0–34.9		1.21 (0.45–3.24)	
				≥35.0		1.85 (0.59–5.85)	
		Physical activity (hours per week)		Continuous	CVD	0.83 (0.70–0.98)	
				No activity	CVD	1.0 (ref)	
				0–1.9		0.52 (0.22–1.23)	
				2.0–4.9		0.38 (0.15–1.00)	
				≥5.0		0.29 (0.08–1.04)	
		Alcohol (drinks per week)		Continuous	CVD	1.01 (0.94–1.08)	
				Non-drinker	CVD	1.0 (ref)	
				0–1.9		0.68 (0.29–1.60)	
				2.0–6.9		1.22 (0.47–3.14)	
				≥7.0		0.49 (0.13–1.86)	
		Smoking		Non-smoker	CVD	1.0 (ref)	
				Former smoker		0.96 (0.43–2.15)	
				Current smoker		2.07 (0.84–5.11)	
		*Post-diagnosis behaviors*					Pre-diagnosis health behavior and demographic, clinical, tumor, and treatment characteristics
		BMI		Continuous	CVD	0.96 (0.85–1.08)	
				18.5–24.9	CVD	1.0 (ref)	
				25.0–29.9		0.90 (0.32–2.51)	
				30.0–34.9		0.63 (0.15–2.70)	
				≥35.0		0.36 (0.05–2.74)	
		Physical activity (hours per week)		Continuous	CVD	1.04 (0.91–1.18)	
				No activity	CVD	1.0 (ref)	
				0–1.9		0.35 (0.04–2.97)	
				2.0–4.9		0.42 (0.05–3.60)	
				≥5.0		2.27 (0.40–12.76)	
		Alcohol (drinks per week)		Continuous	CVD	0.90 (0.67–1.22)	
				Non-drinker	CVD	1.0 (ref)	
				0–1.9		1.43 (0.37–5.62)	
				2.0–6.9		1.53 (0.24–9.89)	
				≥7.0		0.57 (0.04–8.52)	
		Smoking		Non-smoker	CVD	1.0 (ref)	
				Former smoker		0.92 (0.41–2.08)	
				Current smoker		1.27 (0.22–6.86)	

*AJCC* American Joint Committee on Cancer, *AMI* acute myocardial infarction, *BMI* body mass index; kg/m^2^, *CHD* Coronary heart disease, *CI* confidence interval, *CT* chemotherapy, *CVA* cerebrovascular accident, *CVD* cardiovascular disease, *DCIS* ductal carcinoma in situ, *HR* hazard ratio, *HT* hormonal therapy, *MR* mortality ratio, *ref* reference category, *RT* radiotherapy, *SD* standard deviation, *SEER* Surveillance, Epidemiology, and End Results, *US* United States

Riihimäki et al. [5] included all 3,676,472 female Swedish residents born before 1977 (Table 2). Of these, 122,217 were diagnosed with primary invasive breast cancer between 1987 and 2006. During a maximum follow-up of 19 years, 10.4% and 7.5% of women died of CVD, respectively. Women with breast cancer had a 1.14 (95% CI 1.10–1.19) times higher risk to die of coronary heart disease, a 1.29 (95% CI 1.22–1.37) times higher risk to die of heart failure, and a 1.24 (95% CI 1.17–1.32) times higher risk to die of other heart disease than women from the general population, independent of age, socioeconomic index, and geographical region of residence in Sweden (Table 3).

### Cardiovascular disease mortality in breast cancer patients by patient, tumor, and treatment characteristics

Colzani et al. [18] included 12,850 Swedish women younger than 75 years of age at diagnosis with primary invasive stage I to III breast cancer between 1990 and 2006 (Table 2). During a maximum follow-up of ten years, 1.8% of all women died of CVD. After adjusting for clinical, tumor, and treatment characteristics, except the one of interest, women with breast cancer were at increased risk of CVD mortality if they were older at diagnosis (65–74 years vs. 45–54 years: hazard ratio (HR) = 17.9, 95% CI 8.0–39.7), if diagnosed in an earlier calendar period (1990–1994 vs. 2000–2006: HR = 2.1, 95% CI 1.2–3.6), and treated with only surgery (HR = 2.1, 95% CI 1.2–3.8) or surgery in combination with hormonal therapy (HR = 2.2, 95% CI 1.5–3.2) compared with surgery in combination with radiotherapy and hormonal therapy (Table 3).

Hooning et al. [25] included 7425 women younger than 71 years of age at diagnosis with primary invasive stage I to IIIA breast cancer in the Netherlands between 1970 and 1986 (Table 2). During a median follow-up of 13.8 years, 5.3% of all women died of CVD. After adjusting for clinical, tumor, and treatment characteristics, women with breast cancer were at increased risk of CVD mortality with each year increase in age at diagnosis (HR = 1.12, 95% CI 1.10–1.14), if diagnosed in an earlier calendar period (1976–1980 vs. 1981–1986: HR = 1.54, 95% CI 1.11–2.14), and treated with a combination of surgery and radiotherapy compared with only surgery (HR = 2.03, 95% CI 1.33–3.10) (Table 3).

### Cardiovascular disease mortality in breast cancer patients by laterality of the tumor

Bouchardy et al. [20] included 1245 women with a mean age of 57.4 years at diagnosis with primary lymph node-negative breast cancer in Switzerland between 1980 and 2004 (Table 2). During a mean follow-up of 7.7 years, 2.2% of all women died of CVD. Among women treated with radiotherapy, an inner quadrant tumor was associated with a 2.46 (95% CI 1.13–5.37) higher risk of dying of CVD, adjusted for clinical, tumor, and treatment characteristics (Table 3).

Darby et al. [23] included 308,861 women between 20 and 79 years of age at diagnosis with primary in situ or invasive breast cancer in the USA between 1973 and 2001 (Table 2). During a maximum follow-up of 29 years, 4.2% of all women died of CVD. In women treated with radiotherapy and diagnosed between 1973 and 1982, left-sided breast cancer led to higher mortality ratios (MR) ten to 14 years post diagnosis (unadjusted MR = 1.42, 95% CI 1.11–1.82) and over 15 years post diagnosis (unadjusted MR = 1.58, 95% CI 1.29–1.95) compared with right-sided breast cancer (Table 3). More than ten years post diagnosis, women with left-sided breast cancer had a higher risk of death from CVD (unadjusted MR = 1.44, 95% CI 1.26–1.65), acute myocardial infarction (unadjusted MR = 1.43, 95% CI 1.10–1.87), and other ischemic CVD (unadjusted MR = 1.60, 95% CI 1.26–2.02) compared with women with right-sided breast cancer.

Giordano et al. [24] included 24,785 women with primary in situ or invasive breast cancer diagnosed in the US between 1973 and 1988 (Table 2). Mean age at diagnosis was 56.9 years (standard deviation (SD) = 13.2) at diagnosis. Eight years post diagnosis, women with left-sided breast cancer who were diagnosed in 1979 had a (unadjusted) 1.50 (95% CI 1.15–1.87) times higher risk to die of CVD compared with women with right-sided breast cancer diagnosed in the same year (Table 3).

Haque et al. [26] included 140,914 women of all ages with ductal carcinoma in situ (DCIS) in the US between 1973 and 2002 (Table 2).The median follow-up was 11.5 years (interquartile range = 6.8–15.1). Among women diagnosed between 1973 and 1982, a left-sided tumor was associated with a 1.30 (95% CI 1.18–1.42) higher risk of dying of CVD than a right-sided tumor, independent of clinical, tumor, and treatment characteristics. This association was not found for women diagnosed in a more recent calendar period.

Merzenich et al. [21] included 11,982 women with a mean age of 59 years (range = 18–101) at primary diagnosis of in situ or invasive breast cancer in Germany between 1998 and 2008 (Table 2) [21]. During a median follow-up of 6.5 years (range = 0–15), 2.3% of all women died of CVD. Women with left-sided breast cancer did not have a higher risk of dying of CVD than women with right-sided breast cancer, irrespectively of radiotherapy treatment (Table 3). Among women treated with radiotherapy, women with a history of CVD had a 1.73 times (95% CI 1.11–2.68) higher risk of dying of CVD than women without a history of CVD.

### CVD mortality in breast cancer patient by ethnic origin

Berkman et al. [6] included 54,518 white and 6113 black women over 40 years of age at diagnosis with primary DCIS in the US between 1978 and 2010 (Table 2). During a median follow-up of 9.2 years, 6.0% of all women died of CVD. Among women diagnosed with breast cancer between 1990 and 2010, black women had a (unadjusted) 6.43 (95% CI 3.61–11.45) times higher risk of death from CVD compared to white women (Table 3). Unadjusted HRs of CVD death in black compared to white women decreased with increasing age at diagnosis: 9.83 (95% CI 4.56–21.17), 3.35 (95% CI 2.14–5.24), 2.13 (95% CI 1.65–2.74), and 1.07 (95% CI 0.93–1.23) for women of ages 40–49, 50–59, 60–69, and ≥ 70 years, respectively.

Solanki et al. [17] included 462,005 non-Hispanic white and 44,531 Asian and Pacific Islander women diagnosed with breast cancer in the US between 1991 and 2001 (Table 2). Median age at breast cancer diagnosis was 61.2 years (SD = 13.7) for non-Hispanic white women and 56.3 years (SD = 13.1) for Asian and Pacific Islander women. The median follow-up for non-Hispanic white women was 4 years (range = 2-6), during which 5.5% of women died of CVD. The median follow-up for Asian and Pacific Islander women was 3 years (range 2–5), during which 2.6% of women died of CVD. After adjusting for patient, tumor, and registry characteristics, Asian and Pacific Islander women with breast cancer had a HR of 0.77 (95% CI 0.71–0.83) for death from CVD compared to non-Hispanic white women with breast cancer (Table 2). Furthermore, US born Asian and Pacific Islander women with breast cancer had a 1.29 (95% CI 1.08–1.54) times higher risk of death from CVD compared to non-US born Asian and Pacific Islander women with breast cancer.

### CVD mortality in breast cancer patients by diet, body weight, and health-behaviors

McCullough et al. [16] included 4452 women diagnosed with primary invasive breast cancer in Switzerland between 1992 and 2011 who had scored their diet according to the American Cancer Society (AMC) guidelines before breast cancer diagnosis, and of these, 2152 women scored their diet also at least one year after breast cancer diagnosis (Table 2). The AMC guidelines recommend following the general food-based guidelines for primary cancer prevention, which includes eating a plant-based diet rich in vegetables and fruits, whole grains, and which is limited in red and processed meats [27]. Mean age at diagnosis was 70.7 years (SD = 7.2). During a mean follow-up of 9.8 years (SD = 4.9), 5.2% of all women died of CVD. After adjusting for tumor, treatment, and patient characteristics, both pre-diagnostic and post-diagnostic higher AMC diet scores, indicating an unhealthier diet, were not associated with a higher risk of CVD mortality following breast cancer compared with the lowest diet score category (0–2), indicating a healthier diet (Table 3).

Nichols et al. [15] included 5791 women with primary in situ or invasive breast cancer diagnosed in the US between 1988 and 1999 (Table 2). Mean age at diagnosis was 58.4 years (SD = 10.0). During a mean follow-up of 6.4 years (SD = 1.2), 1.6% of all women died of CVD. After correcting for age, menopausal status, and CVD risk factors, Nichols et al. found a 4.15 (95% CI 1.44–12.0) and 2.45 (95% CI 1.46–4.11) times higher risk of death from CVD in women with a pre-diagnosis underweight (body mass index (BMI; kg/m^2^): <18.5) and obesity (BMI: ≥ 30), respectively, compared with women with a pre-diagnosis normal weight (BMI: 18.5–24.9) (Table 3).

Veal et al. [22] included 1925 women aged between 20 and 74 years at diagnosis of DCIS in the US between 1997 and 2006 (Table 2). During a mean follow-up of 6.7 years, 1.8% of all women died of CVD. More hours per week of physical activity before the breast cancer diagnosis was associated with 0.83 (95% CI 0.70–0.98) lower risk of dying of CVD (Table 3).

## Discussion

In this review, we systematically summarized the evidence on the risk and risk factors of death from CVD following breast cancer.

The absolute risk of dying of CVD following breast cancer ranges from 1.6% [15] to 10.4% [5], and the risk of CVD mortality is higher in women with breast cancer than women from the general population [5, 8]. Older age at diagnosis [6, 18, 19, 25], left-sided tumor [23, 24, 26], diagnosis in an earlier calendar period [18, 25], and black ethnic origin [6] are risk factors of CVD mortality following breast cancer.

Several mechanisms are proposed for the increased risk of CVD mortality in women with breast cancer. CVD risk factors, such as obesity and diabetes, may be more present among breast cancer survivors than women from the general population as breast cancer and CVD have shared risk factors [28]. Also, cardiotoxic effects of breast cancer treatments, specifically mediastinal and left-sided radiotherapy, anthracycline-based chemotherapy, and trastuzumab, are well documented to increase the risk of CVD [29–32].

In the current review, studies with longer follow-up, i.e. over 10 years [5, 6, 8], reported higher absolute risks of CVD mortality. The risk of CVD increases with time since diagnosis probably due to increasing age and cardiotoxicity of breast cancer treatments that become apparent after several years [29]. Age is a well-known risk factor for CVD [33–36], and therefore, expected to be found as a risk factor in women with breast cancer [6, 18, 19, 25]. Schonberg et al. [14] found that 26–40% of older women diagnosed with early-stage breast cancer died of CVD, indicating that the risk of CVD is high in specific subgroups and particularly in older women.

The association between left-sided breast cancer and radiotherapy treatment with a higher risk of CVD mortality was found among women diagnosed in the early 1980’s [23, 24, 26]. Radiotherapy treatment was more cardiotoxic in these years as it usually involved higher doses with large irradiation fields irradiating parts of the heart [37, 38]. This may also explain the increased risk of CVD mortality among breast cancer patients diagnosed in an earlier calendar period [18, 25]. Colzani et al. [18] did not find an increased risk of CVD mortality among women treated with radiotherapy and/or chemotherapy. Although the baseline risk of CVD was not reported, this result is probably due to patient selection, i.e. women who did not undergo radiotherapy and/or chemotherapy probably had a higher risk of CVD at baseline. The lower risk of CVD in Asian populations [17] and the higher risk of CVD in black populations [6] are reported by several studies and can be explained by the lower and higher presence of CVD risk factors, respectively, such as high blood pressure, obesity, and lipid levels [39–42].

The present systematic review shows that there are only a limited number of studies investigating the risk and risk factors of CVD mortality following breast cancer, and that these studies are heterogeneous in design, study population, and length of follow-up. Also, the determinants and outcomes, in terms of CVD risk factors and death due to CVD, respectively, vary. We acknowledge that, due to the heterogeneous designs of the included studies, we were unable to perform a meta-analysis, which limited the strength of evidence. Besides limitations, the current review has strengths. This is the first study that systematically summarized the literature on the risk and risk factors of death from CVD following breast cancer. Furthermore, the current systematic review includes a large variety of risk factors of death from CVD in women with breast cancer.

To conclude, the combination of high breast cancer incidence, improved breast cancer survival, presence of CVD risk factors, and cardiotoxic breast cancer treatments has resulted into many breast cancer survivors at risk of CVD. Therefore, it is important to understand the incidence and etiology of CVD in these survivors. Furthermore, identification of women with breast cancer at high risk of CVD is important to minimize the number of women suffering and/or dying of CVD after breast cancer treatment and improve quality of life and long-term prognosis. Clinicians should be able to identify breast cancer patients at increased risk of CVD and provide accurate recommendations for CVD risk reduction strategies specifically for breast cancer survivors at high risk of CVD. The current systematic review, in combination with a recent guideline by Armenian et al. [29] on the prevention and monitoring of cardiac dysfunction in survivors of adult cancers, may help clinicians with such a recommendation. In addition, there are studies investigating the identification of women with breast cancer at high risk of CVD using other measurements, for example, by measuring the coronary artery calcification on radiotherapy planning computed tomography scans [43]. This may further help clinicians with identification of breast cancer patients at high risk of CVD. Identification of breast cancer patients at high risk of CVD is important to optimize CVD prevention of (irreversible) cardiac damage, by adjusting breast cancer treatments accordingly and initializing CVD (preventative) treatment. Furthermore, a tailored individual approach with early and late monitoring of cardiac dysfunction in breast cancer survivors should be implemented in routine care [44].
